# Future world cancer death rate prediction

**DOI:** 10.1038/s41598-023-27547-x

**Published:** 2023-01-06

**Authors:** Oleg Gaidai, Ping Yan, Yihan Xing

**Affiliations:** 1 Shanghai Ocean University, Shanghai, China; 2 University of Stavanger, Stavanger, Norway

**Keywords:** Cancer, Diseases, Risk factors, Mathematics and computing

## Abstract

Cancer is a worldwide illness that causes significant morbidity and death and imposes an immense cost on global public health. Modelling such a phenomenon is complex because of the non-stationarity and complexity of cancer waves. Apply modern novel statistical methods directly to raw clinical data. To estimate extreme cancer death rate likelihood at any period in any location of interest. Traditional statistical methodologies that deal with temporal observations of multi-regional processes cannot adequately deal with substantial regional dimensionality and cross-correlation of various regional variables. Setting: multicenter, population-based, medical survey data-based biostatistical approach. Due to the non-stationarity and complicated nature of cancer, it is challenging to model such a phenomenon. This paper offers a unique bio-system dependability technique suited for multi-regional environmental and health systems. When monitored over a significant period, it yields a reliable long-term projection of the chance of an exceptional cancer mortality rate. Traditional statistical approaches dealing with temporal observations of multi-regional processes cannot effectively deal with large regional dimensionality and cross-correlation between multiple regional data. The provided approach may be employed in numerous public health applications, depending on their clinical survey data.

## Introduction

The National Cancer Institute defines cancer as a group of disorders in which aberrant cells may proliferate and invade neighbouring tissue. Cancer may develop in most regions of the body, resulting in various cancer forms, as indicated below, and can sometimes spread via the blood and lymph systems.

Cancer's statistical characteristics received much attention from the current scientific community ^1–8^. Using current theoretical statistical methods ^9–15^, it is often rather challenging to compute realistic biological system dependability factors and outbreak probability under actual cancer settings. Typically, this results from many degrees of system freedom and random variables driving vastly dispersed dynamic biological systems. In theory, the dependability of a complex biological system may be precisely evaluated using sufficient observations or direct Monte Carlo simulations. Beginning in 1990, however, a portion of the available cancer observation numbers are limited^16–21^. Motivated by the latter point, the authors have developed a unique dependability technique for biological and health systems to forecast and control cancer epidemics more precisely. The whole globe was selected because of the enormous internet health observations and associated research^1^.

In health and engineering fields, statistical modelling of lifetime data and extreme value theory (EVT) are widespread. For example, Gumbel utilised EVT to predict the demography of distinct communities in^20–23^. Recent papers arguing for and against the upper bounds distribution of life expectancy were done by^24^. Often, papers in these fields presume a parametric bivariate lifetime distribution obtained from the exponential distribution to get statistically relevant data^24^. In^25^, the author proposes a new approach that uses Power Variance Function copulas (e.g., Clayton, Gumbel and Inverse Gaussian copulas), conditional sampling, and numerical approximation used in survival analysis. While in a paper by^26^, the authors explain that EVT has been used to predict mutation in evolutionary genetics and further develop a likelihood framework from EVT that was used to determine the fitness effects of the mutation.

Similarly, in^27^, The author applies a Beta-Burr distribution to this EVT hypothesis to calculate the fitness impact. While in^28^, the author presents a bivariate logistic regression model, which was afterwards used to access multiple MS fatalities with walking difficulties and in a cognitive experiment for visual identification. Finally^3^, is a relevant work utilising EVT to evaluate the chance of a global cancer breakout. In^22,23^, similarly, researchers employed EVT to predict and identify cancer abnormalities.

In this research, a cancer outbreak is seen as an unanticipated occurrence that may occur in any location of a nation at any moment; hence, the spatial spread is considered. Moreover, a specific non-dimensional factor $\lambda$ is introduced to forecast the cancer risk at any given time and location. Environmental impacts on biological systems are ergodic. The second possibility is to see the process as reliant on specific external characteristics whose time-dependent change may be modelled as an ergodic process on its own. The incidence data of cancer in one hundred ninety-five world countries during the years 1990–2019 were retrieved from the public website^1^, considered a multi-degree-of-freedom (MDOF) spatio-temporal dynamic bio-system with highly inter-correlated regional components/dimensions.

**Figure 1:**
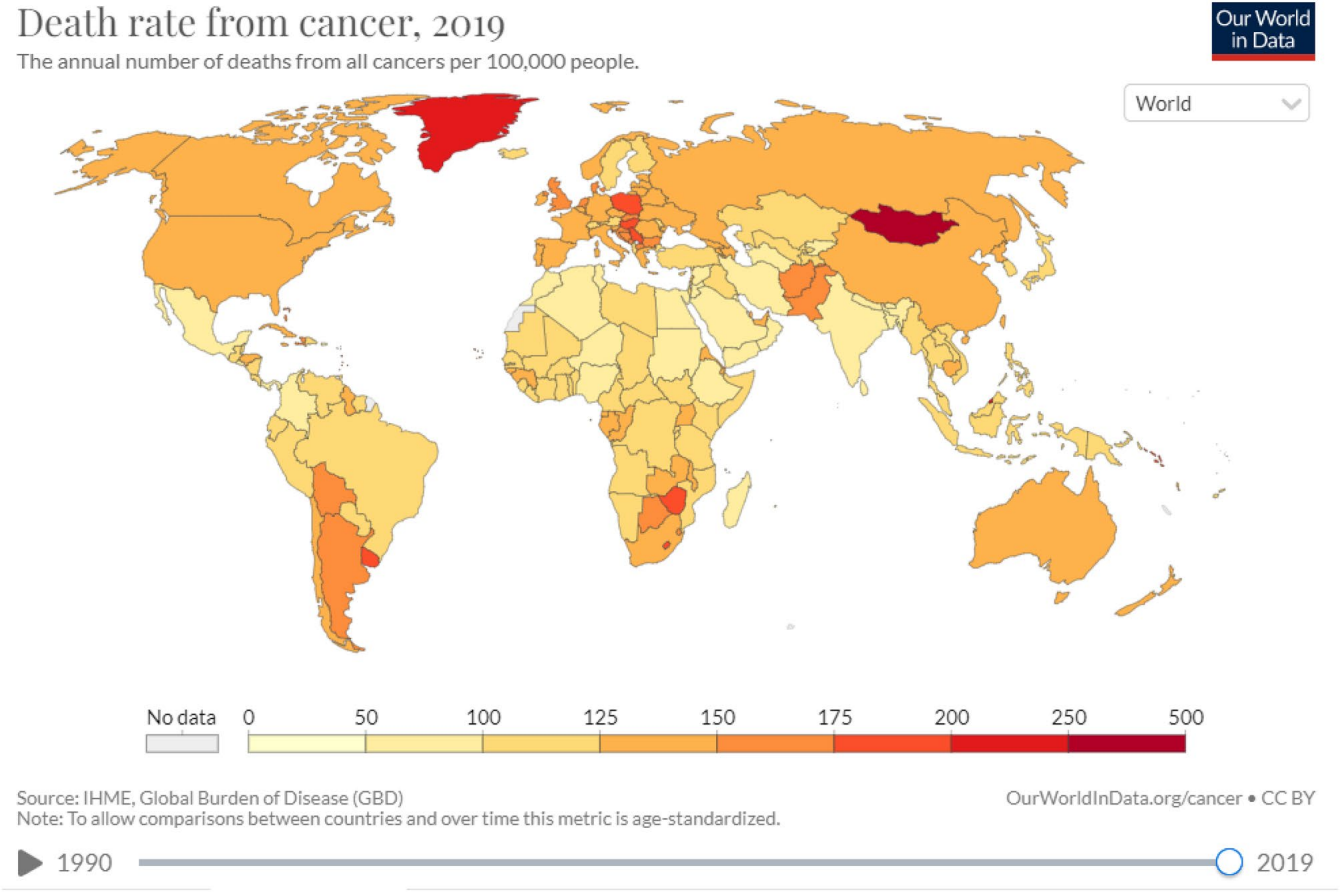
Map of the world with countries and cancer deaths. All world countries were studied in this paper^1^.

This research tries to reduce the danger of future cancer outbreaks by forecasting them. However, it focuses simply on the yearly number of documented patient deaths and not on the symptoms themselves. Figure 1 presents the map of the world's countries.

Further research should incorporate one of the common complexity measures, such as fractal, attractor/embedding dimension, and entropy.

## Methods

Consider an MDOF (multi-degree of freedom) structure subjected to random ergodic environmental factors (stationary in time). The second possibility is to see the process as reliant on certain external characteristics whose time-dependent change may be modelled as an ergodic process on its own. The MDOF biomedical response vector process ${\varvec{R}}\left(t\right)=\left(X\left(t\right), Y\left(t\right), Z\left(t\right), \dots \right)$ is measured and/or simulated over a sufficiently long time interval $(0,T)$. Unidimensional global maxima over the duration of time $(0,T)$ are denoted as ${X}_{T}^{\mathrm{max}}=\underset{0\le t\le T}{\mathrm{max}}X\left(t\right)$, ${Y}_{T}^{\mathrm{max}}=\underset{0\le t\le T}{\mathrm{max}}Y\left(t\right)$, ${Z}_{T}^{\mathrm{max}}=\underset{0\le t\le T}{\mathrm{max}}Z\left(t\right), \dots$.By sufficiently long time $T$ one primarily means a large value of $T$ with respect to the dynamic system auto-correlation time^33–40^.

Let ${X}_{1},\dots ,{X}_{{N}_{X}}$ be consequent in time local maxima of the process $X(t)$ at monotonously increasing discrete time instants ${t}_{1}^{X}<\dots <{t}_{{N}_{X}}^{X}$ in $(0,T)$. The analogous definition follows for other MDOF response components $Y\left(t\right), Z\left(t\right), \dots$ with ${Y}_{1},\dots ,{Y}_{{N}_{Y}};$ ${Z}_{1},\dots ,{Z}_{{N}_{Z}}$ and so on. For simplicity, all ${\varvec{R}}\left(t\right)$ components, and therefore its maxima are assumed to be non-negative. The aim is to estimate the system failure probability

1 $$1-P=\mathrm{Prob}({X}_{T}^{\mathrm{max}}>{\eta }_{X} \cup {Y}_{T}^{\mathrm{max}}>{\eta }_{Y} \cup {Z}_{T}^{\mathrm{max}}>{\eta }_{Z} \cup \dots )$$

with

2 $$P=\underset{\left(0, 0, 0, , \dots \right)}{\overset{\left({\eta }_{X}, {\eta }_{Y}, {\eta }_{Z }, \dots \right)}{\iiint }}{p}_{{X}_{T}^{\mathrm{max}}, { Y}_{T}^{\mathrm{max}}, { Z}_{T}^{\mathrm{max}} , \dots }\left({X}_{T}^{\mathrm{max}}, {Y}_{T}^{\mathrm{max}},{ Z}_{T}^{\mathrm{max}}, \dots \right)d{X}_{T}^{\mathrm{max}}d{Y}_{{N}_{Y}}^{\mathrm{max}}d{Z}_{{N}_{z}}^{\mathrm{max}}\dots$$

being the probability of non-exceedance for response components ${\eta }_{X}$, ${\eta }_{Y}$, ${\eta }_{Z}$,… critical values; $\cup$ denotes logical unity operation; and ${p}_{{X}_{T}^{\mathrm{max}}, { Y}_{T}^{\mathrm{max}}, { Z}_{T}^{\mathrm{max}} , \dots }$ being joint probability density of the global maxima over the entire time span $(0,T)$.

In practice, it is not possible to accurately estimate the latter joint probability distribution ${p}_{{X}_{T}^{\mathrm{max}}, { Y}_{T}^{\mathrm{max}}, { Z}_{T}^{\mathrm{max}} , \dots }$ due to its high dimensionality and available data set limitations. In other words, the time instant when either $X\left(t\right)$ exceeds ${\eta }_{X}$, or $Y\left(t\right)$ exceeds ${\eta }_{Y}$, or $Z\left(t\right)$ exceeds ${\eta }_{Z}$, and so on, the system being regarded as immediately failed. Fixed failure levels ${\eta }_{X}$, ${\eta }_{Y}$, ${\eta }_{Z}$,…are of course individual for each unidimensional response component of ${\varvec{R}}\left(t\right)$. ${X}_{{N}_{X}}^{\mathrm{max}}=\mathrm{max }\{{X}_{j}\hspace{0.17em};j=1,\dots ,{N}_{X}\}={X}_{T}^{\mathrm{max}}$, ${Y}_{{N}_{Y}}^{\mathrm{max}}=\mathrm{max }\{{Y}_{j}\hspace{0.17em};j=1,\dots ,{N}_{Y}\}={Y}_{T}^{\mathrm{max}}$,${Z}_{{N}_{z}}^{\mathrm{max}}=\mathrm{max }\{{Z}_{j}\hspace{0.17em};j=1,\dots ,{N}_{Z}\}={Z}_{T}^{\mathrm{max}}$, and so on.

Next, the local maxima time instants $\left[{t}_{1}^{X}<\dots <{t}_{{N}_{X}}^{X}; {t}_{1}^{Y}<\dots <{t}_{{N}_{Y}}^{Y}; {t}_{1}^{Z}<\dots <{t}_{{N}_{Z}}^{Z}\right]$ in monotonously non-decreasing order are sorted into one single merged time vector ${t}_{1}\le \dots \le {t}_{N}$.Note that ${t}_{N}=\mathrm{max }\{{t}_{{N}_{X}}^{X}, {t}_{{N}_{Y}}^{Y}, { t}_{{N}_{Z}}^{Z}, \dots \}$, $N={N}_{X}+{N}_{Y}+{ N}_{Z}+ \dots$. In this case ${t}_{j}$ represents local maxima of one of MDOF bio-system response components either $X\left(t\right)$ or $Y\left(t\right)$, or $Z\left(t\right)$ and so on. That means that having ${\varvec{R}}\left(t\right)$ time record, one just has to continually and concurrently screen for local maximums of unidimensional response components and record their exceeding the MDOF limit vector $\left({\eta }_{X}, {\eta }_{Y}, {\eta }_{Z},...\right)$ in any of its components $X, Y, Z, \dots$. The maxima of local unidimensional response components are blended into a non-decreasing temporal vector $\overrightarrow{R}=\left({R}_{1}, {R}_{2}, \dots ,{R}_{N}\right)$ in accordance with the merged time vector ${t}_{1}\le \dots \le {t}_{N}$. That is to say, each local maxima ${R}_{j}$ is the actual encountered local maxima corresponding to either $X\left(t\right)$ or $Y\left(t\right)$, or $Z\left(t\right)$ and so on. Finally, the unified limit vector $\left({\eta }_{1}, \dots ,{\eta }_{N}\right)$ is introduced with each component ${\eta }_{j}$ is either ${\eta }_{X}$, ${\eta }_{Y}$ or ${\eta }_{Z}$ and so on, depending on which of $X\left(t\right)$ or $Y\left(t\right)$, or $Z\left(t\right)$ etc., corresponding to the current local maxima with the running index $j$.

Next, a scaling parameter $0<\lambda \le 1$ is implemented to artificially lower limit values for all response components concurrently, namely the new MDOF limit vector $\left({\eta }_{X}^{\lambda },{ \eta }_{Y}^{\lambda }, {\eta }_{z}^{\lambda },...\right)$ with ${\eta }_{X}^{\lambda }\equiv \lambda \cdot { \eta }_{X}$, $\equiv \lambda \cdot { \eta }_{Y}$, ${\eta }_{z}^{\lambda }\equiv \lambda \cdot { \eta }_{Z}$, … is introduced. The unified limit vector $\left({\eta }_{1}^{\lambda }, \dots ,{\eta }_{N}^{\lambda }\right)$ is introduced with each component ${\eta }_{j}^{\lambda }$ is either ${\eta }_{X}^{\lambda }$, ${\eta }_{Y}^{\lambda }$ or ${\eta }_{z}^{\lambda }$ and so on. The latter automatically defines probability $P\left(\lambda \right)$ as a function of $\lambda$, note that $P\equiv P\left(1\right)$ from Eq. (1). Non-exceedance probability $P\left(\lambda \right)$ can be now estimated as follows

3 $$\begin{aligned} P\left( \lambda \right){ } & = {\text{Prob}}\left\{ {R_{N} \le \eta_{N}^{\lambda } , \ldots ,R_{1} \le \eta_{1}^{\lambda } } \right\} \\ & = {\text{Prob}}\{R_{N} \le \eta_{N}^{\lambda } {|} R_{N - 1} \le \eta_{N - 1}^{\lambda } , \ldots ,R_{1} \le \eta_{1}^{\lambda } \} \cdot {\text{Prob}}\left\{ {R_{N - 1} \le \eta_{N - 1}^{\lambda } , \ldots ,R_{1} \le \eta_{1}^{\lambda } } \right\} \\ & = \left( {\mathop \prod \limits_{j = 2}^{N} {\text{Prob}}\{ R_{j} \le \eta_{j}^{\lambda } | R_{j - 1} \le \eta_{1j - }^{\lambda } , \ldots ,R_{1} \le \eta_{1}^{\lambda } \} } \right) \cdot {\text{Prob}}\left( {R_{1} \le \eta_{1}^{\lambda } } \right) \\ \end{aligned}$$

In practice, a dependency between neighbouring ${R}_{j}$ is not always negligible; thus, the following one-step (called here conditioning level $k=1$) memory approximation is introduced

4 $$\mathrm{Prob}\{{R}_{j}\le {\eta }_{j}^{\lambda } |{ R}_{j-1}\le {\eta }_{j-1}^{\lambda },\dots ,{R}_{1}\le {\eta }_{1}^{\lambda }\}\approx \mathrm{Prob}\{{R}_{j}\le {\eta }_{j}^{\lambda } |{ R}_{j-1}\le {\eta }_{j-1}^{\lambda }\}$$

for $2\le j\le N$ (called here conditioning level $k=2$). The approximation introduced by Eq. (4) can be further expressed as

5 $$\mathrm{Prob}\{{R}_{j}\le {\eta }_{j}^{\lambda } |{ R}_{j-1}\le {\eta }_{j-1}^{\lambda },\dots ,{R}_{1}\le {\eta }_{1}^{\lambda }\}\approx \mathrm{Prob}\{{R}_{j}\le {\eta }_{j}^{\lambda } |{ R}_{j-1}\le {\eta }_{j-1}^{\lambda }, {R}_{j-2}\le {\eta }_{j-2}^{\lambda }\}$$

where $3\le j\le N$ (will be called conditioning level $k=3$), and so on. The goal is to monitor each isolated failure that occurs locally first in time, thereby preventing cascade local inter-correlated exceedances.

Equation (5) presents subsequent refinements of the statistical independence assumption. The latter type of approximation enables capturing the statistical dependence effect between neighbouring maxima with increased accuracy. Since the original MDOF bio-process ${\varvec{R}}\left(t\right)$ was assumed ergodic and therefore stationary, the probability $p_{k} \left( \lambda \right){\text{: = Prob }}\{R_{j} > \eta _{j}^{\lambda } ~|~R_{{j - 1}} \le \eta _{{j - 1}}^{\lambda } ,~~R_{{j - k + 1}} \le \eta _{{j - k + 1}}^{\lambda } \}$ for $j\ge k$ will be independent of $j$ but only dependent on conditioning level $k$. Thus non-exceedance probability can be approximated as in the Naess-Gaidai method^29,30^, where

6 $${P}_{k}(\lambda )\approx \mathrm{exp }(-{N\cdot p}_{k}\left(\lambda \right))\hspace{0.17em} , k\ge 1$$

Note that Eq. (6) follows from Eq. (1) by neglecting $\mathrm{Prob}({R}_{1}\le {\eta }_{1}^{\lambda })\approx 1$, as the design failure probability is usually very small. Further, it is assumed $N "k$. Note that Eq. (5) is similar to the well-known mean up-crossing rate equation for the probability of exceedance^32^. There is obvious convergence with respect to the conditioning parameter $k$

7 $$P=\underset{k\to \infty }{\mathrm{lim}}{P}_{k}(1); p\left(\lambda \right)=\underset{k\to \infty }{\mathrm{lim}}{p}_{k}\left(\lambda \right)$$

**Figure 2:**
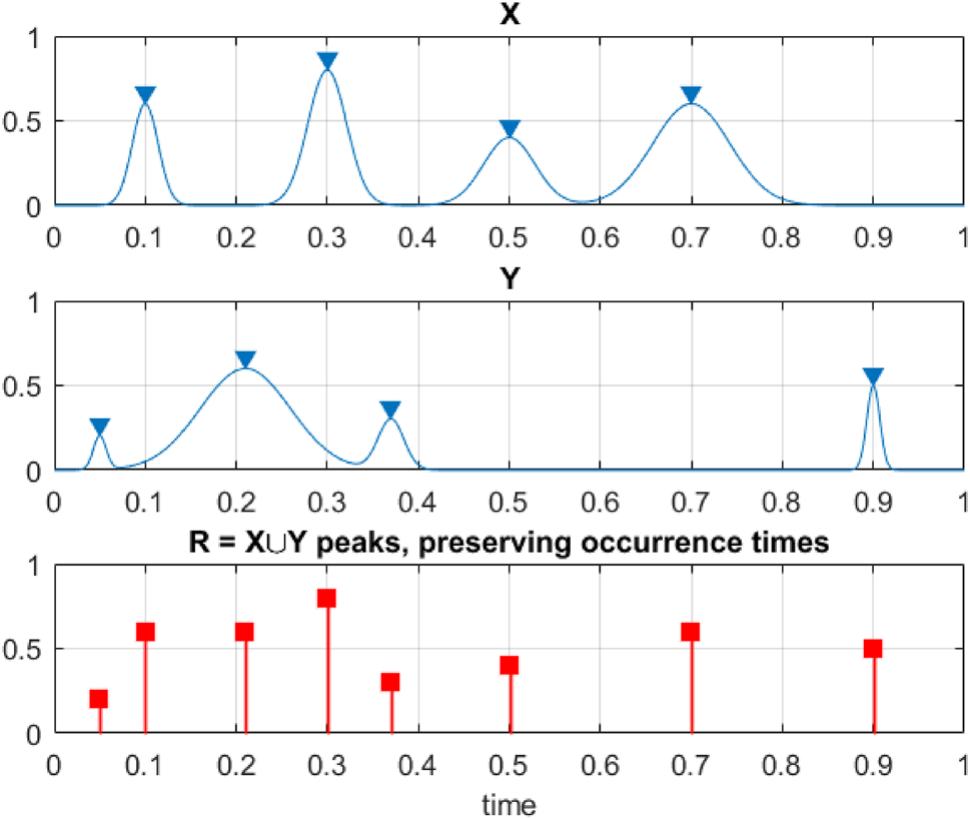
Example of how two example processes, X and Y, are merged to create a new synthetic vector $\overrightarrow{R}$.

Note that Eq. (6) for $k=1$ turns into the quite well-known non-exceedance probability relationship with the mean up-crossing rate function

8 $$P\left(\lambda \right) \approx \mathrm{exp }(-{\nu }^{+}(\lambda )\hspace{0.17em}T); {\nu }^{+}\left(\lambda \right)={\int }_{0}^{\infty }\zeta {p}_{R\dot{R}}\left(\lambda ,\zeta \right)d\zeta$$

where ${\nu }^{+}(\lambda )$ is the mean up-crossing rate of the response level $\lambda$ for the above assembled non-dimensional vector $R\left(t\right)$ assembled from scaled MDOF bio-system response $\left(\frac{X}{{\eta }_{X}}, \frac{Y}{{\eta }_{Y}}, \frac{Z}{{\eta }_{Z}}, \dots \right)$. Note that constructed $\overrightarrow{R}$-vector has no data loss at all; see Fig. 2.

In the preceding, the assumption of stationarity has been employed. The proposed methodology can also treat the non-stationary case. An illustration of how the methodology can be used to treat non-stationary cases is provided. Consider a scattered diagram of $m=1,..,M$ environmental states, each short-term bio-environmental state having a probability ${q}_{m}$, so that $\sum_{m=1}^{M}{q}_{m}=1$. The corresponding long-term equation is then

9 $${p}_{k}(\lambda )\equiv \sum_{m=1}^{M}{p}_{k}(\lambda ,m){q}_{m}$$

with ${p}_{k}(\lambda ,m)$ being the same function as in Eq. (7) but corresponding to a specific short-term environmental state with the number $m$. The above introduced ${p}_{k}(\lambda )$ as functions are often regular in the tail, specifically for values of $\lambda$ approaching and exceeding $1$. More precisely, for $\lambda \ge {\lambda }_{0}$, the distribution tail behaves similarly to ${\text{exp}}\left\{-{\left(a\lambda +b\right)}^{c}+d\right\}$ with $a, b, c, d$ being suitably fitted constants for suitable tail cut-on ${\lambda }_{0}$ value. Therefore, one can write

10 $${p}_{k}(\lambda )\approx {\text{exp}}\left\{-{\left({a}_{k}\lambda +{b}_{k}\right)}^{{c}_{k}}+{d}_{k}\right\}, \lambda \ge {\lambda }_{0}$$

Next, by plotting ${\text{ln}}\left\{{\text{ln}}\left({p}_{k}(\lambda )\right)-{d}_{k}\right\}$ versus ${\text{ln}}\left({a}_{k}\lambda +{b}_{k}\right)$, often nearly perfectly linear tail behaviour is observed. Optimal values of the parameters ${a}_{k}, {b}_{k}, {c}_{k},{p}_{k},{q}_{k}$ may also be determined using a sequential quadratic programming (SQP) method incorporated in the NAG Numerical Library^31^.

For levels of $\lambda$ approaching $1$, the approximate limits of a *p-*% confidence interval (CI) of ${p}_{k}\left(\lambda \right)$ can be given as follows^41–46^

11 $${\mathrm{CI}}^{\pm }(\lambda )={p}_{k}(\lambda )(1\pm \frac{f(p)}{\sqrt{(N-k+1){p}_{k}(\lambda )}})\hspace{0.17em}.$$

with $f(p)$ being estimated from the inverse normal distribution, for example, $f\left(90\%\right)=1.65$, $f\left(95\%\right)=1.96$. with $N$ being the total number of local maxima assembled in the analysed vector $\overrightarrow{R}$.

## Results

Predictions of cancer-related mortality have been the focus of epidemiology and mathematical biology for a long time. It is common knowledge that the dynamics of public health are a highly non-linear, multidimensional, spatially cross-correlated dynamic system that is always difficult to analyse. Previous studies have used a variety of approaches to model cancer cases. This section presents the application of the above-described methodology to the real-life cancer data sets, presented as a new annual recorded time series for all world countries. The statistical information presented in this section was obtained from the official World website^1^. The website provides cancer death rates per country from 1990 to 2019. Patient death numbers from one hundred ninety-five different world countries were chosen as components $X, Y, Z, ...$, thus constituting an example of a one hundred ninety-five dimensional (195D) dynamic biological system. To unify all 195 measured time series $X, Y, Z,\dots$ the following scaling was performed

12 $$X\to \frac{X}{{\eta }_{X}}, Y\to \frac{Y}{{\eta }_{Y}}, Z\to \frac{Z}{{\eta }_{Z}}, \dots$$

making all 195 responses non-dimensional and having the same failure limit equal to 1. Failure limits ${\eta }_{X}, {\eta }_{Y}, {\eta }_{Z}, \dots$, or in other words, cancer thresholds, are not an obvious choice. The most straightforward choice would be for different countries to set failure limits equal to the corresponding country population in per cent to local population, basically making $X, Y, Z, \dots$ equal to the annual death rate per country. Next, all local maxima from 195 measured time series were merged into one single time series by keeping them in time non-decreasing order: $\overrightarrow{R}=\left(\mathrm{max}\left\{{X}_{1},{Y}_{1},{Z}_{1},\dots \right\},\dots ,\mathrm{max}\left\{{X}_{N},{Y}_{N},{Z}_{N},\dots \right\}\right)$ with the whole vector $\overrightarrow{R}$ being sorted according to non-decreasing times of occurrence of these local maxima.

**Figure 3:**
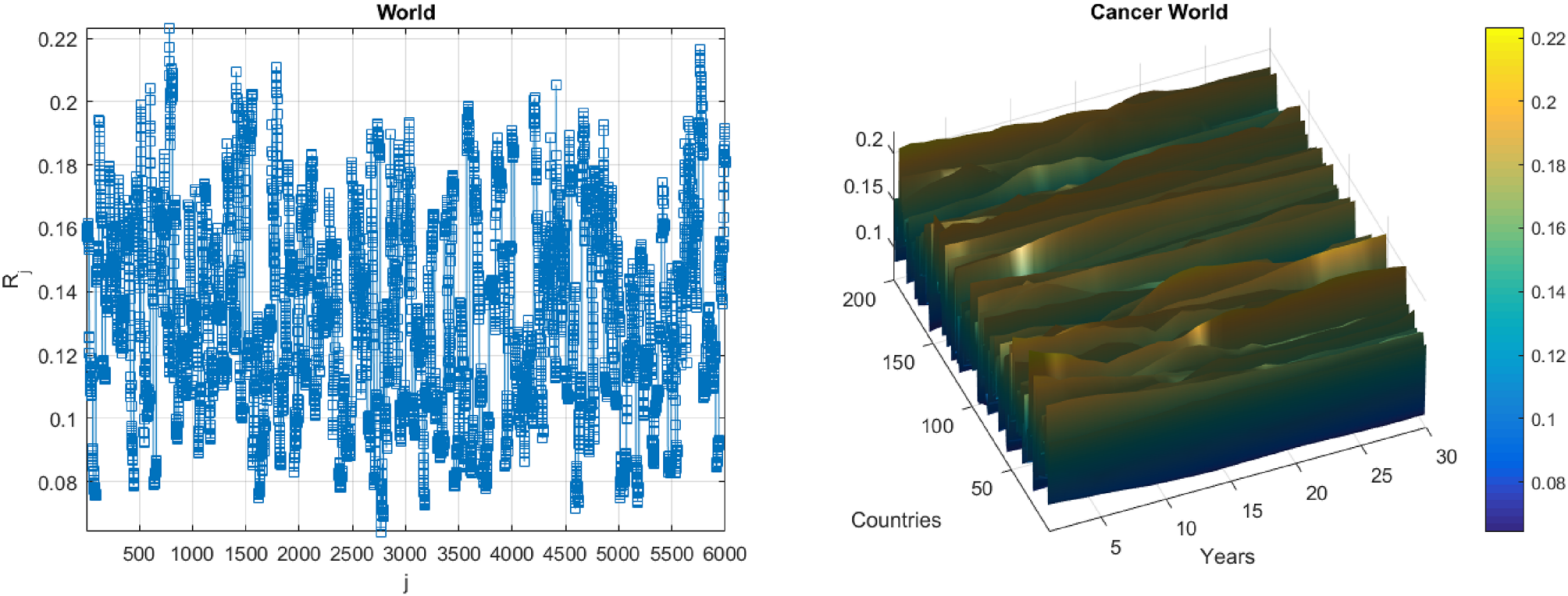
Annual cancer annual death cases. Left: as % of local population per country and year. Right: in per cent as 195D vector $\overrightarrow{R}$. Scaled by Eq. (9) in per cent of the corresponding country population.

Figure 3 presents the number of new annual recorded deaths as a 195D vector $\overrightarrow{R}$, consisting of assembled regional new annual death rate for each corresponding country. Greenland, Mongolia, Monaco and Hungary data were excluded from analysis, since were regarded as outliers. Note that vector $\overrightarrow{R}$ is assembled of different regional components with different cancer backgrounds. Index $j$ is just a running index of local maxima encountered in a non-decreasing time sequence.

**Figure 4:**
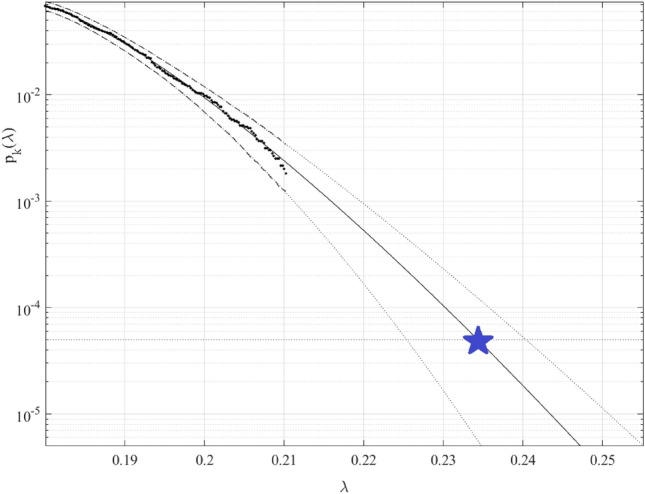
Death rate prediction. 100 years return level extrapolation of ${p}_{k}\left(\lambda \right)$ towards critical level (indicated by a star) in per cent of the local population. Extrapolated 95% CI indicated by dotted lines. Percentage of the local population on the horizontal axis.

Figure 4 presents the annual death rate (percentage of deaths from cancer to the population of a given country) prediction, 100 years return level extrapolation according to Eq. (10) towards cancer outbreak with a 100-year return period, indicated by the horizontal dotted line. Somewhat beyond, $\lambda =0.18$% cut-on value was used, percentage of the local population on the horizontal axis. The dotted lines indicate extrapolated 95% confidence interval according to Eq. (11). According to Eq. (5) $p\left(\lambda \right)$ is directly related to the target failure probability $1-P$ from Eq. (1). Therefore, in agreement with Eq. (5), system failure probability $1-P\approx {1-P}_{k}\left(1\right)$ can be estimated. Note that in Eq. (6), $N$ corresponds to the total number of local maxima in the unified response vector $\overrightarrow{R}$. Conditioning parameter $k=3$ was found to be sufficient due to occurrence of convergence with respect to $k$, see Eq. (6). Figure 4 exhibits reasonably narrow 95% CI. The latter is an advantage of the proposed method.

The predicted cancer death rate in any world country in any year to come for the next 100 years was found to be about 0.24%.

Note that, although being unique, the above-described technique has the distinct benefit of using existing measured data sets very effectively owing to its capacity to deal with the multidimensionality of the health system and to execute correct extrapolation using relatively small data sets.Note that the predicted non-dimensional $\lambda$ level, indicated by the star in Fig. 4, represents the probability of cancer outbreak in any world country in the years to come.

In order to validate the suggested methodology, a twice smaller data set was used to obtain predictions for the same probability levels of interest as in Fig. 4. The twice smaller data set was obtained from the original data set by sampling every second consecutive data point. Predicted $\lambda$, based on reduced data set, was found within 95% CI based on the entire data set, indicated in Fig. 4.

The second-order difference plot (SODP) originated from the Poincare plot. SODP provides observing the statistical situation of consecutive differences in time series data.

**Figure 5:**
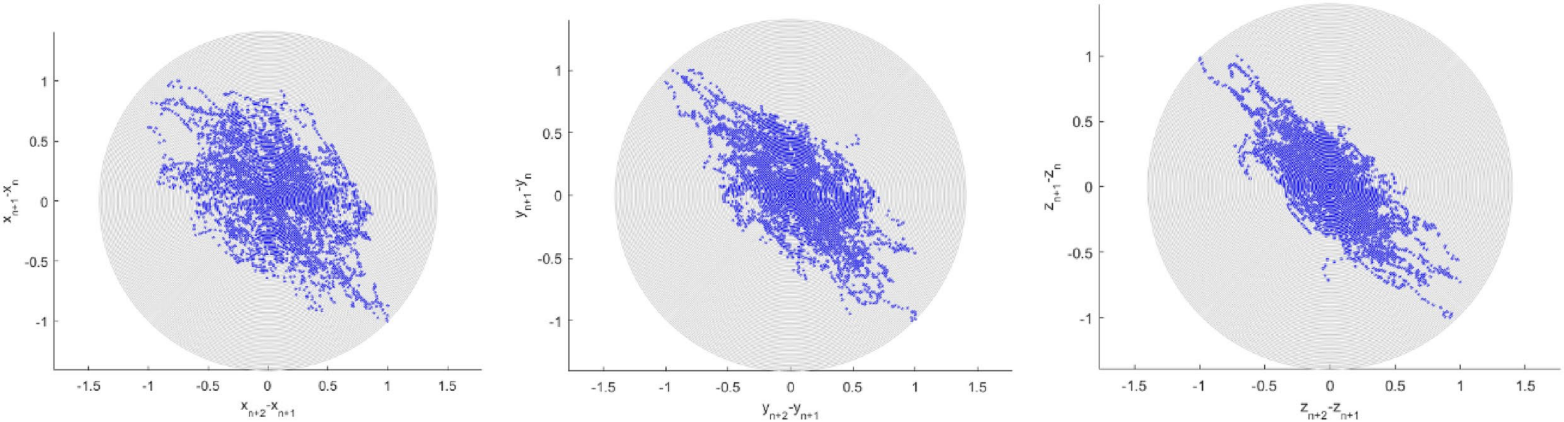
Cancer global statistics. Left: SODP plot. Middle: TODP, Right: FODP.

Figure 5 presents SODP along with a third-order difference plot TODP and a fourth-order difference plot FODP. These kinds of plots can be used for data pattern recognition and comparison with other data sets, for example, for the entropy artificial intelligence (AI) recognition approach^32^. Note that EVT is asymptotic and 1DOF, while this study introduces MDOF and sub-asymptotic approaches. To summarise, the predicted non-dimensional λ level, indicated by the star in Fig. 4, represents the probability of world cancer deaths in the years to come. The methodology's limitation lies in its assumption of the underlying bio-environmental process quasi-stationarity.

## Discussion

Traditional health systems reliability methods dealing with observed time series do not have the advantage of dealing efficiently with systems possessing high dimensionality and cross-correlation between different system responses. The essential advantage of the introduced methodology is its ability to study the reliability of high dimensional non-linear dynamic systems.

Despite the simplicity, the present study successfully offers a novel multidimensional modelling strategy and a methodological avenue to implement forecasting of the cancer death rate. Proper setting of health system alarm limits (failure limits) per country has been discussed.

This paper studied recorded cancer death rates from all world countries, constituting an example of a one hundred ninety-five dimensional (195D) observed from 1990 to 2019. In real-time, the novel reliability method was applied to cancer annual death rate numbers as a multidimensional system. The theoretical reasoning behind the proposed method is given in detail. Note that the use of direct either measurement or Monte Carlo simulation for dynamic biological system reliability analysis is attractive; however, dynamic system complexity and its high dimensionality require the development of novel robust and accurate techniques that can deal with a limited data set at hand, utilising available data as efficient as possible.

The main conclusion is that the public health system under local environmental and epidemiologic conditions is well managed. This study predicted an annual death rate 100-year return period risk level equal to about 0.24%. Therefore, under current national health management conditions, cancer still represents a future threat to world health.

This study further aimed to develop a general-purpose, robust, and straightforward multidimensional reliability method. The method introduced in this paper has been previously validated by application to a wide range of simulation models, but for only one-dimensional system responses and, in general, very accurate predictions were obtained. Both measured and numerically simulated time series responses can be analysed. It is shown that the proposed method produced a reasonable confidence interval. Thus, the suggested methodology may become appropriate for various non-linear dynamic biological systems reliability studies. Finally, the suggested methodology can be used in many public health applications. The presented cancer example does not limit areas of new method applicability (Supplementary file).

The suggested method can work well with non-stationary data sets (for example, seasonal variations) as soon as they represent the proof of interest. If, however, there is an underlying trend in the process of interest or the data was manipulated, those effects have to be identified. In that case, trend analysis should be performed, a topic for future studies. In any case, authors assume that within 3 years, horizon quasi-stationarity may be assumed. Therefore, the limitation of this study lies within the assumption of bio-system quasi-stationarity, which is, of course, not valid for many years to come.

## Supplementary Information

Supplementary Information 1.

Supplementary Information 2.

## Data Availability

The datasets analysed during the current study are available online^1^ https://ourworldindata.org/causes-of-death. The authors confirm that all methods were performed following the relevant guidelines and regulations according to the Declarations of Helsinki.

## Code availability

For software used to extrapolate probability tails in this study, see https://github.com/cran/acer.
